# Association of care fragmentation with financial and clinical outcomes of transcatheter aortic valve replacement in the US: A retrospective cohort study

**DOI:** 10.1371/journal.pone.0353361

**Published:** 2026-07-21

**Authors:** Arjun Chaturvedi, Saad Mallick, Nguyen K. Le, Kevin Tabibian, Jeffrey Balian, Dariush Yalzadeh, Barzin Badiee, Radoslav Zinoviev, Peyman Benharash

**Affiliations:** 1 Cardiovascular Outcomes Research Laboratories (CORELAB), David Geffen School of Medicine, University of California, Los Angeles (UCLA), Los Angeles, California, United States of America; 2 Division of Cardiology, David Geffen School of Medicine, UCLA, Los Angeles, California, United States of America; 3 Department of Surgery, David Geffen School of Medicine, UCLA, Los Angeles, California, United States of America; Taipei Medical University, TAIWAN

## Abstract

**Purpose:**

Care fragmentation (CF), defined as readmission to a non-index facility, has historically been linked with adverse clinical and financial outcomes for a variety of cardiac procedures. However, its impact on patients undergoing transcatheter aortic valve replacement (TAVR) remains understudied.

**Methods:**

This retrospective study investigated the prevalence and impact of CF on outcomes of patients undergoing TAVR. All adults (≥18 years) undergoing an isolated TAVR procedure, who were readmitted within 90 days, were tabulated using the Nationwide Readmissions Database from 2016 to 2021. A 90-day readmission window was chosen in accordance with prior literature on readmission. Patients treated at a non-index facility were categorized into the CF cohort. Multivariable logistic models were developed to characterize the association of care fragmentation status with perioperative complications and resource utilization; candidate variables were selected using elastic net regularization to minimize variable collinearity.

**Results:**

Of an estimated 55,891 patients who were readmitted within 90 days, 37.4% were treated at a non-index facility. While TAVR utilization more than doubled, both the readmission and care fragmentation rates fell during the study period. Following adjustment, CF status was associated with respiratory (AOR [Adjusted Odds Ratio] 1.20, 95% CI [Confidence Interval] 1.11–1.29, p < 0.001), gastrointestinal (AOR 1.21, 95% CI 1.05–1.38, p = 0.008), and infectious complications (AOR 1.17, 95% CI 1.10–1.25, p < 0.001) at readmission. Furthermore, CF was linked with greater risk of non-home discharge (AOR: 1.13 95% CI [1.03–1.25], P = 0.01).

**Conclusions:**

As TAVR utilization continues to expand nationwide, further efforts to enhance care coordination and streamline information sharing are warranted to mitigate the burden of care fragmentation.

## Introduction

Transcatheter aortic valve replacement (TAVR) has become widely adopted as an effective treatment strategy for severe symptomatic aortic stenosis, with its indications continuing to expand [1]. In fact, several recent clinical trials have demonstrated comparable short- and long-term outcomes following surgical aortic valve replacement (SAVR) and TAVR across strata of surgical risk [2,3]. These factors, alongside the expansion of TAVR eligibility and increased operator experience, have contributed to a nearly ten-fold increase in procedural volume between 2010 and 2023 [1,4]. Despite such advances including expedited discharge, TAVR readmission rates remain high, with more than 10% of patients being rehospitalized within 30 days [5,6]. Additionally, Kolte and colleagues found that patients readmitted within 30 days following TAVR experienced higher rates of acute kidney injury, major bleeding, and cardiac arrest, compared to others [7].

Care fragmentation (CF), defined as readmission to a non-index facility, may further contribute to the poor clinical and financial outcomes among rehospitalized patients [8]. Prior investigation has cited a multitude of factors including inadequate medical history, variations in center quality, and limited data sharing as potential mechanisms underlying worse outcomes in CF [9]. Existing literature has highlighted the increased healthcare expenditures and inferior clinical outcomes associated with CF across multiple procedural contexts [10]. However, implications of care fragmentation in cardiac populations, remain insufficiently characterized [10,11]. In a study of approximately 9,000 TAVR patients between 2011–2015, Wang and colleagues found that over 5,000 patients experienced care fragmentation and reported CF to be linked with increased mortality and all-cause readmissions at one year [12–17]. However, while technological advancements and increased data sharing were associated with partially mitigating CF, their study relied on decade-old data and was not nationally representative. Given the significant rise in TAVR volume across the US, a contemporary national analysis of the incidence and impact of CF following TAVR procedures is warranted.

In the present work, we examined the association of CF with readmission outcomes after hospitalization in a contemporary cohort of TAVR patients. We hypothesized CF to be associated with greater risk of complications at readmission along with similar resource utilization.

## Methods

This was a retrospective cohort study of the 2016–2021 Nationwide Readmissions Database (NRD). Aggregated from 28 state inpatient databases, the NRD is a nationally representative all-payer database and provides accurate estimates for ~60% of all hospitalizations in the United States. Each record in the NRD is given a unique identifier, allowing for subsequent readmissions to be linked within each state and calendar year.

**Fig 1:**
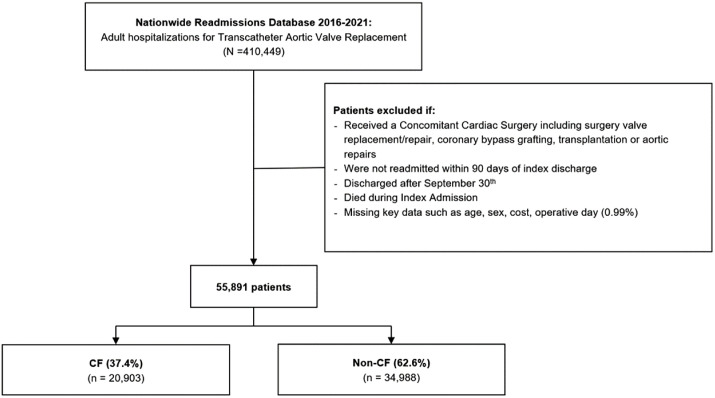
Study CONSORT diagram illustrating the study design and patient groupings. All estimates are based on survey-weighted methodology. Key data include age, sex, costs, in-hospital mortality, elective status, and income. *CF, care fragmentation.*

All adult (≥18 years) hospitalizations for isolated (TAVR) were tabulated in the 2016−2021 Nationwide Readmissions Database (NRD) using previously reported International Classification of Diseases Tenth Revision procedure codes (ICD-10) [1]. While only patients requiring nonelective rehospitalization within 90 days of index discharge were considered, those readmitted to a non-index facility comprised the CF cohort (others non-CF). Our cohort included only patients undergoing first-time TAVR procedures, excluding those who received valve-in-valve procedures due to their distinct risk profile and prior surgical history. Records with concurrent cardiac procedures or missing key data as well as discharges after September 30^th^ of each year, were not considered (Fig 1).

Patient demographics and hospital characteristics including age, sex, income quartile, primary payer, and hospital teaching status were defined in accordance with the Healthcare Cost and Utilization Project dictionary data [18]. Hospitalization costs were estimated by applying HCUP center-specific cost-to-charge ratios to total charges and inflating all values to 2021 dollars using the Personal Health Care Price Index. [19]. Facility-wide cost-to-charge ratios were applied uniformly across payer categories, preventing adjustment for payer-specific reimbursement differences and potentially introducing nondifferential measurement error in estimated hospitalization expenditures. Hospitals were ranked according to the annual institutional TAVR caseload with centers in the top quartile classified as High-Volume Centers (HVH, Others: Low Volume-Centers (LVH)). Non-home discharge was defined as discharge to an acute care hospital, intermediate care facility, or skilled nursing facility.

The previously validated Comorbid Operative Risk Score (CORE score) was employed to numerically capture the burden of chronic conditions [20]. The CORE score employs machine learning algorithms to generate a mortality prediction score based on comorbidities [20]. The presence of specific conditions such as coronary artery disease, chronic pulmonary disease, and coagulopathy was ascertained using previously validated ICD-10 codes [21]. Other comorbidities including obesity, defined as a body mass index greater than 30, and smoking, identified by documentation of nicotine dependence or tobacco use, were similarly captured using validated ICD-10 classifications [21]. Cardiac (myocardial infarction or cardiac arrest), neurological (stroke or transient ischemic attack), infectious (sepsis or deep wound infection), gastrointestinal (bile leak, gastrointestinal bleeding, intestinal perforation, hemoperitoneum, or abdominal fluid collection), thromboembolic (deep vein thrombosis or pulmonary embolism), and respiratory (pneumothorax, acute respiratory distress syndrome, acute respiratory failure, prolonged ventilation >96 hours, pneumonia, or pleural effusion) complications, were similarly identified using previously published ICD-10 codes [21]. Diagnosis related group codes were used to tabulate the primary reason for readmission.

The primary outcome of interest was the development of any in-hospital complication (listed above) at readmission. Secondary endpoints included hospitalization costs, postoperative length of stay, and non-home discharge upon readmission.

An exploratory, post hoc subgroup analysis was performed to assess whether the relationship between care fragmentation and outcomes varies based on institutional procedural experience, given the established volume-outcome relationship in TAVR. Patients within the CF-cohort were stratified into four subgroups based on operative volume of their index hospitalization and readmission. Patients receiving TAVR and subsequently readmitted to high-volume centers comprised the high-high cohort (HH) while those only treated at low volume centers comprised the low-low cohort (LL). Patients initially receiving TAVR at a HVH and then were readmitted to a LVH were grouped as high-low (HL) while those with an index admission at a LVH with subsequent readmission to a HVH comprised the low-high cohort (LH).

Continuous variables are summarized as medians with interquartile range (IQR) or means with standard deviations (SD), while categorical variables are reported as proportions (%). The Adjusted Wald, chi-square, and Mann-Whitney *U* tests were used to compare patient and hospital characteristics where applicable. Cuzick’s nonparametric test was applied to determine the significance of temporal trends (nptrend) [22]. Multivariable logistic regression and generalized linear models were fit to evaluate independent associations between CF and study endpoints. Covariates were selected using elastic net regularization, which minimizes collinearity through a penalized least squares methodology [23]. Model covariates included both patient-level and hospital-level factors. Model performance was evaluated using receiver operating characteristics and calibrations belts, as appropriate. Interaction terms were incorporated into risk-adjusted models to evaluate the effect of institutional TAVR volumes at index admission and readmission in the CF only sub analysis. To contextualize the potential for type II error, we calculated the minimum detectable effect; under a two-sided α = 0.05, 95% power, and 1:3 group allocation, we found 17,400 patients would be required, which is well below our realized cohort. Regression outputs are reported as adjusted odds ratio (AOR) or beta coefficients (β) for logistic and linear models with 95% confidence intervals (CI), respectively. An α of 0.05 was set for statistical significance. All statistical analysis was performed on Stata 16.1 software (StataCorp, LLC, College Station, TX). This study was deemed exempt from full review by the Institutional Review Board at University California, Los Angeles.

## Results

**Fig 2:**
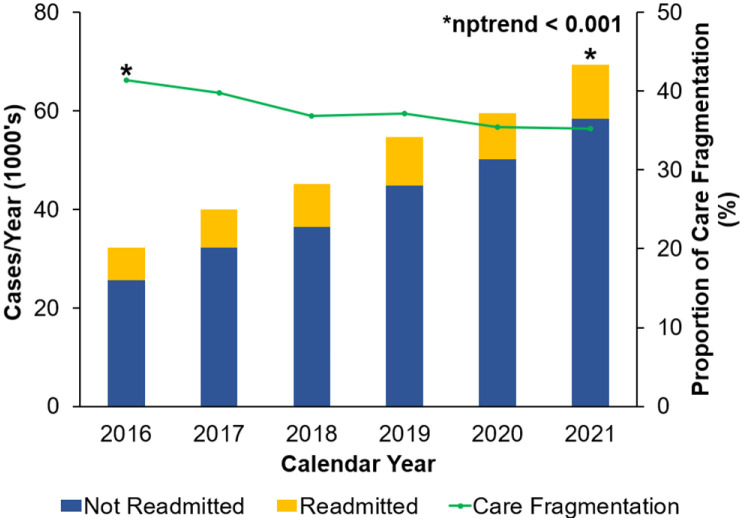
Annual trends in Transcatheter Aortic Valve Replacement utilization and Care Fragmentation rates. The rate care fragmentation decreased significantly over the study period, nptrend < 0.001.

Of an estimated 300,842 TAVR hospitalizations performed during the study period, 55,891 (17.6%) entailed a nonelective readmission within 90 days of index discharge, 37.4% of which (n = 20,903) were at a non-index facility (CF). The total number of TAVR procedures more than doubled during the study period (32,216 procedures in 2016 vs 69,398 in 2021, nptrend<0.001; Fig 2). Additionally, rates of both 90-day readmissions (20.7 in 2016 vs 15.7% in 2021, P < 0.01), and care fragmentation rates (41.4 in 2016 vs 35.3% in 2021, P = 0.01) decreased significantly (Fig 2).

Subjects in the CF cohort were slightly older (80.0 ± 8.5 vs 79.0 ± 8.4 years, P < 0.01) and more likely to be female (46.5 vs 45.2%, P < 0.01; Table 1), compared to others. The CF group also presented with a higher median CORE score (44 [30–59] vs 43 units [28–58], P < 0.01) and greater frequency of coronary artery disease (64.4 vs 63.3%, P < 0.01), coagulopathy (14.2 vs 12.7%, P < 0.01), and smoking (40.4 vs 38.2%, P < 0.01; Table 1). The groups were comparable in regards to insurance coverage and income distribution (Table 1).

**Table 1:** Demographic, clinical, and hospital characteristics by care fragmentation status.

	*Non-CF* (n = 33,112)	*CF* (n = 20,903)	*P-value*
**Patient Characteristics**			
Age (years, mean ± SD)	80.0 ± 8.5	79.0 ± 8.4	<0.01
Female (%)	45.2	46.4	<0.01
Corescore (median [IQR])	43 [29–58]	44 [30–59]	<0.01
			
*Income Quartile (%)*			0.09
76th - 100th	23.6	24.8	
51st - 75th	27.8	26.5	
26th - 50th	27.2	28.0	
0–25th	21.4	20.7	
*Payer Status (%)*			0.6
Private	5.3	5.4	
Medicare	91.4	91.2	
Medicaid	1.3	1.5	
Uninsured/other	2.0	1.9	
*Comorbidities (%)*			
Coronary Artery Disease	63.3	64.7	0.02
Hypertension	91.0	91.5	0.21
Atherosclerosis	23.0	24.1	0.08
Diabetes	42.6	43.4	0.2
Chronic Pulmonary Disease	31.3	33.2	<0.01
Acute Kidney Infection	44.5	45.0	0.5
Liver Disease	4.3	4.7	0.2
Coagulopathy	12.7	14.2	<0.01
Anemia	12.8	14.3	<0.01
Obesity	22.0	21.1	0.13
Smoking	38.2	40.4	<0.01
*Hospital teaching status (%)*			0.02
Non-Metropolitan	1.3	0.8	
Metropolitan Non-Teaching	11.2	9.7	
Metropolitan Teaching	87.5	89.5	

Data are presented as mean ± standard deviation, median [interquartile range], or percentage, as appropriate.

Care fragmentation (CF) was defined as receipt of transcatheter aortic valve replacement at a hospital different from the index hospitalization; non-CF indicates care at the same hospital.

CORE score represents a composite comorbidity burden derived from NRD variables.

Continuous variables were compared using the adjusted Wald test or Mann–Whitney U test, as appropriate; categorical variables were compared using the chi-square test.

Statistical significance was defined as a two-sided p value < 0.05.

*CF, care fragmentation; IQR, interquartile range; SD, standard deviation.

After risk adjustment, several factors were associated with greater odds of CF, including an increasing CORE score (AOR: 1.12 per unit 95% CI [1.08–1.15], P < 0.01) and age (AOR: 1.01 per year 95% CI [1.01–1.01], P < 0.01). Additionally, higher center volume (AOR: 0.99 per case 95% CI [0.98–0.99], P < 0.01) at index admission was associated with reduced odds of care fragmentation. However, income and primary payer status did not appear to alter the risk of CF (Table 2).

**Table 2:** Factors associated with care fragmentation following transcatheter aortic valve replacement.

	AOR/β Coefficient (with 95% CI)	*P-value*
**Demographics and Hospital Characteristics (AOR)**		
Age	1.01 [1.01–1.01]	<0.01
Female Sex	1.02 [0.97–1.08]	0.49
Core Score	1.12 [1.08–1.15]	<0.01
Annual Volume	0.99 [0.98–0.99]	<0.01
**Income Quartile (AOR)**		
76th – 100th quartile (Reference)		
51st – 75th quartile	0.92 [0.85–1.03]	0.06
26th – 50th quartile	1.02 [0.93–1.10]	0.67
0–25th quartile	0.96 [0.87–1.06]	0.44
**Primary Payer (AOR)**		
Private (Reference)		
Medicare	1.13 [0.98–1.24]	0.09
Medicaid	1.21 [0.87–1.45]	0.24
Uninsured/Other Payer	1.34 [0.93–1.72]	0.08

Results are reported as adjusted odds ratios (AOR) or β coefficients with 95% confidence intervals (CI), as appropriate.

Multivariable regression models included fixed effects for hospital and patient characteristics.

Model covariates included age, sex, income quartile, primary payer status, annual transcatheter aortic valve replacement volume, CORE score, hospital teaching status, and relevant comorbidities.

Income quartile and primary payer categories are reported relative to the indicated reference groups.

Statistical significance was defined as p value < 0.05.

*AOR, adjusted odds ratio; CI, confidence interval; IQR, interquartile range.

On unadjusted analysis, CF patients experienced greater rates of neurological (7.4 vs 6.0%, P < 0.01), respiratory (17.9 vs 16.0%, P < 0.01), gastrointestinal (4.1 vs 3.5%, P < 0.001), and infectious (22.6 vs 19.4%, P < 0.001) complications upon readmission, compared to their non-CF counterparts (Table 3). However, CF patients exhibited reduced rates of cardiac (10.8 vs 12.3%, P < 0.01) and thromboembolic (2.3 vs 2.7%, P = 0.05) complications. Additionally, the CF cohort had longer postoperative hospital stays at index admission (3.8 ± 4.9 vs 3.7 days ± 4.9, P = 0.004) as well as greater rates of nonhome discharge (25.5 vs 21.5%, P < 0.01), compared to the non-CF cohort. Hospitalization costs were similar between the groups as shown in Table 2.

**Table 3:** Unadjusted outcomes upon readmission following transcatheter aortic valve replacement, by care fragmentation status.

	*Non-CF* (n = 33,112)	*CF* (n = 20,903)	*P-value*
**Clinical Outcomes**			
Complications (%)			
Neurological	6.0	7.4	<0.01
Thromboembolic	2.7	2.3	0.04
Cardiac	12.3	10.8	<0.01
Respiratory	16.0	17.9	<0.01
Gastrointestinal	3.5	4.1	0.02
Infectious	19.4	22.6	<0.01
**Financial Outcomes** LOS (days, mean ± SD)	3.7 ± 4.9	3.8 ± 4.9	<0.01
Costs ($1,000s, mean ± SD)	56.9 ± 30.4	57.3 ± 32.2	0.48
Nonhome discharge (%)	21.5	25.5	<0.01

Data are presented as mean ± standard deviation or percentage, as appropriate.

P-values were calculated using the chi-square test for categorical variables and the adjusted Wald test for continuous variables.

Statistical significance was defined as a p value < 0.05.

*CF, care fragmentation; LOS, length of stay; SD, standard deviation.

After risk-adjustment, care fragmentation remained independently associated with increased odds of respiratory (AOR: 1.20 95% CI [1.11–1.29], P < 0.01), gastrointestinal (AOR: 1.21 95% CI [1.05–1.38], P = 0.01), and infectious (AOR: 1.17 95% CI [1.10–1.25], P < 0.01) complications during readmission (Table 4).

**Table 4:** Adjusted outcomes upon readmission following transcatheter aortic valve replacement, by care fragmentation status.

	AOR/β Coefficient (with 95% CI)	*P-value*
**Clinical outcomes**		
Complications (AOR)		
Neurological	1.07 [0.96–1.19]	0.25
Thromboembolic	0.89 [0.75–1.06]	0.20
Cardiac	0.98 [0.89–1.07]	0.58
Respiratory	1.20 [1.11–1.29]	<0.01
Gastrointestinal	1.21 [1.05–1.38]	<0.01
Infectious	1.17 [1.10–1.25]	<0.01
**Resource Utilization**		
LOS (β, days)	−0.09 [−0.21 - + 0.04]	0.16
Costs (β, $1,000s)	−0.33 [−1.24 - + 0.59]	0.48
Nonhome discharge (AOR)	1.13 [1.03 - 1.25]	<0.01

Results are reported as adjusted odds ratios (AOR) or β coefficients with 95% confidence intervals (CI), as appropriate.

Multivariable regression models were adjusted for patient- and hospital-level characteristics, including age, sex, income quartile, primary payer status, annual transcatheter aortic valve replacement volume, CORE score, hospital teaching status, and relevant comorbidities.

Length of stay and cost outcomes were modeled as continuous variables, while clinical outcomes and nonhome discharge were modeled as binary outcomes.

The reference category for all comparisons was non–care fragmentation.

Statistical significance was defined as a two-sided p value < 0.05. The variance inflation factor was 1.33 for this model.

*AOR, adjusted odds ratio; CI, confidence interval; LOS, length of stay.

**Fig 3:**
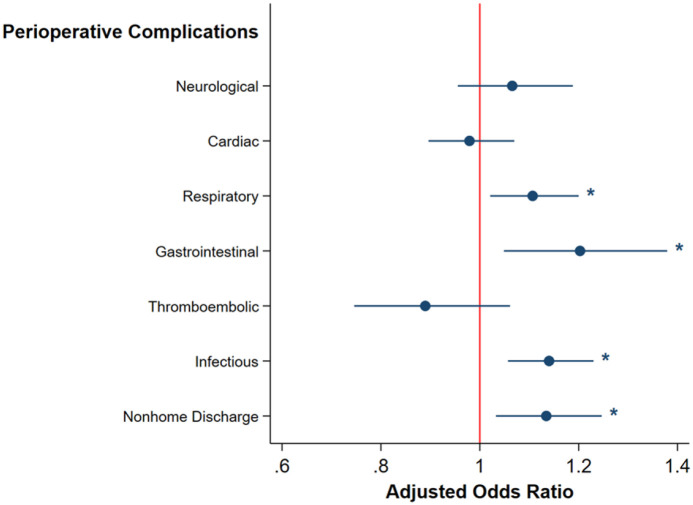
Association of care fragmentation status with selected outcomes at readmission, with non-care fragmentation as reference.

Additionally, CF was linked to higher odds of nonhome discharge (AOR: 1.13 95% CI [1.03–1.25], P = 0.01). However, CF appeared not to be significantly associated with LOS (β: −0.09 days 95% CI [−0.21 – + 0.04], P = 0.16) or aggregate inpatient facility costs captured in the NRD (β: -$327 95% CI [−1240 – + 35], P = 0.48; Fig 3).

An additional analysis was performed on elderly (≥80 years) TAVR recipients to assess the impact of care fragmentation within a more homogenous cohort. Following risk adjustment with non-CF as reference, care fragmentation was independently associated with greater odds of respiratory (AOR: 1.24, 95% CI [1.10–1.42], P < 0.01), infectious (AOR: 1.19, 95% CI [1.09–1.30], P < 0.01), and gastrointestinal complications (AOR: 1.16, 95% CI [1.06–1.39], P < 0.01) during readmission. Moreover, CF was linked to higher rates of nonhome discharge, but comparable hospitalization costs and LOS compared to their non-CF counterparts (Table 5).

**Table 5:** Adjusted outcomes upon readmission among patients aged ≥80 years following transcatheter aortic valve replacement, by care fragmentation status.

	AOR/β Coefficient (with 95% CI)	*P-value*
**Clinical outcomes**		
Complications (AOR)		
Neurological	1.05 [0.92–1.21]	0.47
Thromboembolic	0.89 [0.75–1.06]	0.20
Cardiac	1.01 [0.90–1.14]	0.88
Respiratory	1.24 [1.10–1.42]	<0.01
Gastrointestinal	1.16 [1.06–1.39]	<0.01
Infectious	1.19 [1.09–1.30]	<0.01
**Resource Utilization**		
LOS (β, days)	−0.08 [−0.25 – + 0.08]	0.34
Costs (β, $1,000s)	−0.66 [−2.20 – + 0.88]	0.40
Nonhome discharge (AOR)	1.15 [1.03–1.30]	0.02

Results are reported as adjusted odds ratios (AOR) or β coefficients with 95% confidence intervals (CI), as appropriate.

Multivariable regression models were adjusted for patient- and hospital-level characteristics, including age, sex, income quartile, primary payer status, annual transcatheter aortic valve replacement volume, CORE score, hospital teaching status, and relevant comorbidities.

Length of stay and cost outcomes were modeled as continuous variables, while clinical outcomes and nonhome discharge were modeled as binary outcomes.

The reference category for all comparisons was non–care fragmentation.

Statistical significance was defined as a two-sided p value < 0.05.

*AOR, adjusted odds ratio; CI, confidence interval; LOS, length of stay.

A subgroup analysis was performed to examine the association of hospital volume on index admission and subsequent non-index hospitalization for the CF cohort. Relative to the HH cohort, LL was associated with increased odds of respiratory (AOR: 1.31, 95% CI [1.01–1.71], P = 0.04), gastrointestinal (AOR: 1.67, 95% CI [1.23–2.70], P < 0.01), and infectious (AOR: 1.23, 95% CI [1.09–1.52], P = 0.01) complications upon readmission (Table 6). The HL and LH cohorts noted no differences in developing perioperative complications compared to their HH counterparts. Additionally, the LL cohort experienced prolonged LOS (β: + 2.75 days, 95% CI [+0.42 – + 5.07], P = 0.02) while both the LL (β: + $7,787, 95% CI [+4,298 – + 11,275], P < 0.01) and HL (β: + $5,895, 95% CI [+2,945 – + 8,846], P < 0.01) cohorts were associated with greater hospital expenditure compared to their HH counterparts. Non-home discharge rates remained similar across all cohorts. However, formal interaction testing between index and readmission hospital volumes revealed no significant interaction effect on either financial or clinical outcomes.

**Table 6:** Adjusted outcomes upon readmission among care-fragmented patients following transcatheter aortic valve replacement, stratified by hospital volume category.

	*HL*	*95% CI*	*P-value*	*LH*	*95% CI*	*P-value*	*LL*	*95% CI*	*P-value*
**Clinical Outcomes (AOR)**									
Neurological	0.87	0.61–1.22	0.41	0.45	0.11–1.88	0.27	0.72	0.51–1.06	0.08
Thromboembolic	0.91	0.55–1.51	0.72	1.88	0.37–2.92	0.45	0.92	0.55–1.55	0.75
Cardiac	1.04	0.81–1.35	0.74	0.99	0.43–2.31	0.98	1.07	0.82–1.40	0.61
Respiratory	1.11	0.86–1.44	0.42	0.52	0.24–1.62	0.23	1.31	1.01–1.71	0.04
Gastrointestinal	1.36	0.85–2.17	0.21	1.09	0.78–1.45	0.91	1.67	1.23–2.70	0.01
Infectious	1.10	0.90–1.36	0.35	1.36	0.66–2.83	0.40	1.23	1.09–1.52	0.01
**Financial Outcomes**									
Length of stay, days (β)	−0.02	−5.07 – + 5.01	0.99	+4.62	−3.18 – + 9.65	0.25	+2.75	+0.92– + 10.75	0.02
Non-home discharge (AOR)	0.86	0.65–1.13	<0.001	1.09	0.73–1.78	0.88	0.88	0.73–0.94	<0.01
Costs, $1,000 USD (β)	+5.9	−0.91 – + 9.83	0.27	−1.9	−8.76 – + 6.70	0.82	+7.79	−2.35 – + 12.45	0.38

Results are reported as adjusted odds ratios (AOR) or β coefficients with 95% confidence intervals (CI), as appropriate.

Models were restricted to care-fragmented patients and were stratified by hospital volume category. HH denotes the reference category.

Clinical outcomes and nonhome discharge were modeled as binary outcomes; length of stay and costs were modeled as continuous outcomes.

Multivariable models were adjusted for patient- and hospital-level characteristics, including age, sex, income quartile, primary payer status, annual transcatheter aortic valve replacement volume, CORE score, hospital teaching status, and relevant comorbidities.

Statistical significance was defined as a two-sided p value < 0.05.

* AOR, adjusted odds ratio; CI, confidence interval; HH, high–high; HL, high–low; LH, low–high; LL, low–low; LOS, length of stay.

## Discussion

Care fragmentation has been associated with inferior clinical outcomes across several medical conditions but remains understudied in TAVR [10,11]. In this national analysis, TAVR utilization more than doubled between 2016 and 2021, while both care fragmentation and readmission rates declined. Older age and higher comorbidity burden were associated with increased odds of fragmented care, whereas treatment at high-volume centers was associated with lower fragmentation rates. Fragmented care was associated with increased odds of respiratory, gastrointestinal, and infectious complications during readmission. Because interaction testing between index and readmission hospital volume was not significant, volume-stratified findings should be interpreted cautiously and considered hypothesis-generating.

Congruent with previous literature, we found the 90-day TAVR readmission rates to significantly decline between 2016 and 2021 [24]. This observation, in part, may be explained through advances in clinician experience, better patient selection, and use of lower profile TAVR platforms, obviating the need to surgical vascular access and general anesthesia [1,25,26]. An additional mechanism for the decline in TAVR readmissions may be the utilization of TAVR in lower risk patients as described by Waksman and colleagues [3,27]. Despite these advances, patients undergoing TAVR continue to experience higher rates of care fragmentation and non-elective readmissions relative to those receiving other transcatheter procedures, such as mitral valve replacement [28]. Van Belle et al. reported TAVR patients to be more likely to be readmitted due to a higher prevalence of undiagnosed transthyretin cardiac amyloidosis, persistent left ventricular hypertrophy, and supraventricular arrhythmias [29]. Emerging strategies such as post-discharge remote monitoring may further improve early detection of complications following TAVR [30].

Fragmented care was associated with increased odds of gastrointestinal, infectious, and respiratory complications during readmission. This association persisted in a subgroup analysis of TAVR recipients over the age of 80, with those receiving fragmented care being associated with greater odds of perioperative complications [31]. Despite the modest magnitude of the observed effect, the consistency of this association across a range of complications may reflect the cumulative impact of care discontinuity. Ineffective care coordination among providers is a key contributor to care fragmentation [8]. Examined primarily within the transition from inpatient to outpatient settings, inadequate inter-provider communication and limited clinical data sharing have been estimated to account for a significant portion of adverse events during the intermediate post-discharge period [9]. TAVR patients are especially susceptible to experiencing fragmented care due to their complex health profiles and advanced age [23]. One potential avenue for reducing readmission and care fragmentation rates involves developing a TAVR care coordinator program. A 2022 pilot study of three German hospitals found the implementation of a TAVR care coordinator to be associated with a reduction in 30-day all cause readmissions, improved patient satisfaction, and stronger care continuity between providers [32]. The addition of a care coordinator may help to shorten the treatment pathway while facilitating better decision-making and streamlined care at non-index hospitals [33]. The use of health information technology interoperability has been further associated with a significant reduction in readmissions and care fragmentation. Li and colleagues found implementing health information technology interoperability led to a reduction in all cause readmissions, throughput time, and interhospital transfers for patients with acute myocardial infarction in New York State [34]. Increasing the utilization of health information interoperability for TAVR patients and incentivizing a widespread exchange of clinical data could improve the efficiency of healthcare delivery while reducing readmissions and hospital resource utilization.

Patients receiving care at high-volume centers demonstrated lower rates of care fragmentation. This effect was also observed even when examining CF patients alone, with those receiving care at low-volume hospitals demonstrating higher rates of complications compared to their counterparts who received the entirety of their care at high volume centers. Despite these directional differences, formal interaction testing between index and readmission hospital volume was not significant, possibly suggesting such associations may reflect residual variation as opposed to true effect modification by hospital volume. Higher volume centers are often speculated to have improved outcomes due to robust discharge planning and care coordination [35]. These trends have been previously observed in TAVR, with Vemulapalli and colleagues noting an inverse relationship between hospital volume and 30-day all-cause readmissions as well as in-hospital mortality for patients undergoing aortic valve replacement [36]. Ando et al. reported patients treated at non-metropolitan, non-teaching centers experienced more than a twofold increase in the likelihood of nonelective readmission compared to those at metropolitan, teaching institutions [37]. The link between metropolitan teaching hospitals, often synonymous with high-volume centers, and reduced odds of readmission may be in part explained by increased hospital staffing coupled with enhanced postoperative patient monitoring and management. Given the demonstrated benefits of treatment at tertiary hospitals, some have called for the centralization of care to high-volume centers in a variety of fields including TAVR [38]. While regionalization of treatment to high-volume TAVR centers may streamline care and reduce operative variability, its impact on increased travel distance and potential adverse consequences towards continuity of care cannot be ignored [39].

There are several important limitations to the present study. The NRD is a retrospective, administrative database subject to variations in coding practices between healthcare providers and institutions. Given the retrospective nature, causal relationships between care fragmentation and outcomes of interest cannot be established. The NRD captures readmissions to participating hospitals and within each calendar year, creating the potential for missed rehospitalizations. The NRD lacks certain granular data such as laboratory values, traditional operative risk assessments such as the Society of Thoracic Surgeons Predicted Risk of Mortality, as well as imaging studies, and aortic valve area and ventricular function. Accordingly, these findings should not be interpreted as evidence of overall episode-of-care cost neutrality, as professional fees and post-discharge expenditures are not captured. Detailed expenditure information, including specific drivers of cost such as ICU stays, radiology fees, and procedural expenses remain unavailable in the NRD, thus limiting comprehensive economic interpretation. Moreover, patient satisfaction in their care at the index hospital is not documented. The NRD does not have data regarding the number of hospitals in each region and does not permit geographic analysis. The NRD only captures inpatient admissions without outpatient encounters or the ability to link patients across multiple providers and health systems. In addition, while care fragmentation was treated as a binary outcome, it may be affected by unmeasured socioeconomic and health system-levels factors not fully captured in the NRD, including variables such as health literacy and community-level resources potentially biasing the observed association. Care fragmentation was operationalized as a binary indicator of readmission to a non-index facility, which may not fully capture the spectrum of multi-institutional care. Under nondifferential misclassification of a binary exposure, observed effect estimates are attenuated toward the null by a factor approximating (sensitivity + specificity − 1). If even a modest proportion of fragmented readmissions were misclassified as non-fragmented due to undetected inter-facility transfers or same-system readmissions, true associations could be meaningfully larger than those reported. Accordingly, observed outcome differences between fragmented and non-fragmented care likely represent conservative estimates of the true effect. Furthermore, the binary operationalization precludes assessment of potential dose-response relationships between the degree of multi-institutional care and readmission outcome severity, as the NRD does not encode the number of distinct facilities involved in a patient’s care pathway or the sequencing of inter-facility transitions. Hospital-level clustering was not explicitly modeled and may have resulted in underestimated variance and residual confounding from between-center heterogeneity. Furthermore, in-hospital mortality during readmission was not treated as a competing risk, such that early death may have limited complication accrual and led to underestimation of complication rates among the most critically ill patients. The subgroup analysis stratifying patients by index and readmission hospital volume may have been underpowered, limiting our ability to detect a true interaction between these variables. Furthermore, defining care fragmentation as a binary metric may oversimplify the complexity of multi-institutional care patterns, particularly in the NRD’s inability to capture outpatient encounters, which could result in the misclassification of fragmented care. Such nondifferential misclassification would be expected to bias effect estimates toward the null and may underestimate the true association between care fragmentation and outcomes. Moreover, a comprehensive sensitivity analysis of unmeasured socioeconomics was not feasible within the NRD due to it lacking individual-level data on income, education, and neighborhood deprivation. The NRD also does not include validated frailty assessments, prosthesis type, procedural access site, or operative complexity metrics. Discharge disposition at the index admission, which may influence readmission site selection, was not available as a model covariate. The absence of clinically relevant variables such as prosthesis type, procedural access site, operative complexity, and discharge disposition may contribute to possible residual confounding, which may not be fully resolved no elastic net covariate selection. Omission of these variables may contribute to residual confounding despite elastic net-guided covariate selection.“ Hospital volume strata was defined using sample-based quartiles, with differences in subgroup estimates possibly reflecting the influence of data-cutoffs. The NRD does not provide sufficient granularity to characterize care fragmentation beyond a binary classification of index versus non-index readmission, precluding evaluation of transfer chains, outpatient encounters, or transition timing between facilities. Consequently, future studies using longitudinal claims data or integrated health information networks are needed to determine whether increasing degrees of fragmentation are associated with progressively worse outcomes. Hospital-level clustering was not formally addressed through multilevel modeling, which may result in underestimation of standard errors and contribute to residual confounding from between-center heterogeneity. Additionally, in-hospital mortality during readmission was not modeled as a competing risk; early death may preclude complication accrual, potentially underestimating true complication rates in the most critically ill patients. Likewise, the NRD does not capture outpatient encounter data, limiting the analysis of multi-institutional care patterns. Finally, the omission of valve-in-valve TAVR procedures may bias and limit the generalizability of these finds to the broader TAVR population. Nonetheless, we used robust statistical methods and the largest all-payer dataset in the United States aro reduce the risk of bias and enhanced the generalizability of our findings.

In summary, care fragmentation appears to be linked with inferior clinical outcomes in patients undergoing TAVR. Notably within the care fragmented cohort, treatment at low-volume TAVR centers was associated with greater resource utilization and complication rates. Given the increasing utilization of TAVR nationally, further exploration of efficient information sharing, care coordination, and health equity policy is warranted to reduce the burden of care fragmentation.
